# The association between physical activity and cardiac performance is dependent on age: the Copenhagen City Heart Study

**DOI:** 10.1007/s10554-019-01566-0

**Published:** 2019-03-01

**Authors:** Gowsini Joseph, Rasmus Mogelvang, Tor Biering-Sørensen, Gitte Nielsen, Peter Schnohr, Peter Sogaard

**Affiliations:** 1 The Copenhagen City Heart Study, Bispebjerg & Frederiksberg Hospital, Copenhagen University, Frederiksberg, Denmark; 2 Department of Clinical Medicine, Aalborg University, Soendre Skovvej 15, 9000 Aalborg, Denmark; 3 Department of Cardiology & Centre for Clinical Research, North Denmark Regional Hospital, Hjorring, Denmark; 4 Department of Cardiology, Rigshospitalet, Copenhagen University, Copenhagen, Denmark; 5 Department of Cardiology, Herlev & Gentofte Hospital, Copenhagen University, Hellerup, Denmark; 6 Department of Cardiology, Aalborg University Hospital, Aalborg, Denmark

**Keywords:** Exercise, Cardiac function, Tissue Doppler imaging, Echocardiography, Population study, Cardiac time intervals, Physical activity

## Abstract

This study aimed to test the hypothesis that regular physical activity is associated with improved cardiac function measured by tissue Doppler imaging (TDI) in the general population. Within a large prospective community-based population study, cardiac function was assessed in 2221 persons by TDI. Longitudinal displacement (LD), early diastolic velocity (e’), and myocardial performance index (MPI) was obtained by TDI. Linear univariable and multivariable regression analyses were performed in relation to age groups (< 50 years, 50–65 years, > 65 years) and self-reported level of physical activity: I (inactivity), II (light activity), III (moderate activity), and IV (high-level activity). Participants < 50 years in the most active group had significantly better cardiac performance when compared to all other activity levels (higher levels of e’, LD, and lower levels of MPI). The findings remained with statistical significance after adjustment for sex, ischemic heart disease, diabetes, hypertension, and body mass index (e’ = 11.0, 95% CI (10.4–11.6), p < 0.001; LD = 12.8 (12.3–13.4), p < 0.003; MPI: 0.40 (0.38–0.42), p = 0.02). In age > 65 years, there was a tendency of impaired cardiac function in higher levels of exercise. Interaction analysis revealed that age significantly modified the association between physical activity and cardiac function (p < 0.001). We found a positive association between higher level of physical activity and improved cardiac function in younger persons (< 50 years). In the general population, however, the association interacted with age and amongst persons above 65 years there was a negative association between higher level of physical activity and cardiac function.

**Electronic supplementary material:**

The online version of this article (10.1007/s10554-019-01566-0) contains supplementary material, which is available to authorized users.

## Introduction

Worldwide, health authorities recommend regular physical activity as a means of improving health. This recommendation is based on multiple studies on the beneficial association between physical activity, mortality, and the development of cardiac disease in patients with known heart disease and the general population [1–10]. However, a recent publication from the Copenhagen City Heart Study has indicated that strenuous physical activity is associated with higher mortality in healthy joggers [11]. Investigations of the association between cardiac performance and activity level in the general population are sparse.

Tissue Doppler imaging (TDI) provides a comprehensive evaluation of systolic and diastolic left ventricular function and is superior to conventional echocardiography for risk assessment in patients with heart disease and the general population [12–16]. Previous investigations have indicated that TDI not only identifies deteriorating cardiac function in the general asymptomatic population, but also provides prognostic information on cardiovascular mortality and morbidity independently of traditional risk factors [14, 17, 18]. Moreover, TDI has been used to document improvement in systolic and diastolic function following targeted dedicated exercise protocols in healthy individuals as well as in patients with cardiovascular risk [19–21]. However, most of these studies have included populations of younger age.

Cardiac time intervals are closely related to cardiac physiology, hemodynamics, and mechanics. Myocardial performance index (MPI), also known as the Tei-index, is a method that combines the systolic and diastolic phases of the cardiac cycle. MPI is calculated by dividing the sum of the isovolumic contraction time (IVCT) and the isovolumic relaxation time (IVRT) by the ejection time (ET). In individuals with impaired cardiac function, IVCT and IVRT are prolonged, and ET might be shortened. Thus, MPI is higher in individuals with heart disease than in healthy subjects. MPI has been reported to be associated with cardiovascular mortality in patients with heart disease and the general population [12, 22, 23].

Using data from the Copenhagen City Heart Study, we explored whether higher levels of habitual physical activity in the general population are associated with improved cardiac function assessed by TDI. We chose to focus on the following parameters: e’ (diastolic performance), longitudinal displacement (LD, systolic performance), and MPI (combined systolic and diastolic function). Since TDI-parameters are highly dependent on age, we decided to study the population in the following three age groups: < 50 years, 50–65 years, and > 65 years.

## Methods

### Study population

The Copenhagen City Heart Study is a prospective cohort study of cardiovascular disease and risk factors among Danish citizens. At the first examination in 1976–1978, a random sample of citizens from a well-defined area of Copenhagen City was invited to participate in the study. This present study used data from the fourth examination in 2001–2003. The population consisted of persons who had been invited to the three previous examinations, and a random sample of persons from the younger age group (n = 1000). In total, 6237 persons participated. A total of 2221 randomly selected persons underwent echocardiography including TDI. Persons with atrial fibrillation, significant valvular stenosis, or regurgitation were excluded (n = 157). Due to missing information on physical activity level, 11 persons were excluded. A total of 2053 persons were included in the current study. All subjects gave informed consent to participate, and the study was performed in accordance with the second Helsinki Declaration and approved by the regional ethics committee.

### Echocardiography

Three experienced echocardiography technicians performed all echocardiogram examinations using GE Vingmed Ultrasound’s Vivid Five with a 2.5-MHz probe (Horten, Norway). All participants were examined with color TDI and two-dimensional and M-mode echocardiography in the left lateral decubitus position. All images were recorded with second-harmonic imaging at the time of end expiration. Collected data were stored on magneto-optical disks on an external hard drive (FireWire, LaCie, France) and analyzed offline with a commercially available software program (EchoPac, GE Medical). The investigator was blinded to other information about the participants.

### Color tissue Doppler imaging

**Fig. 1:**
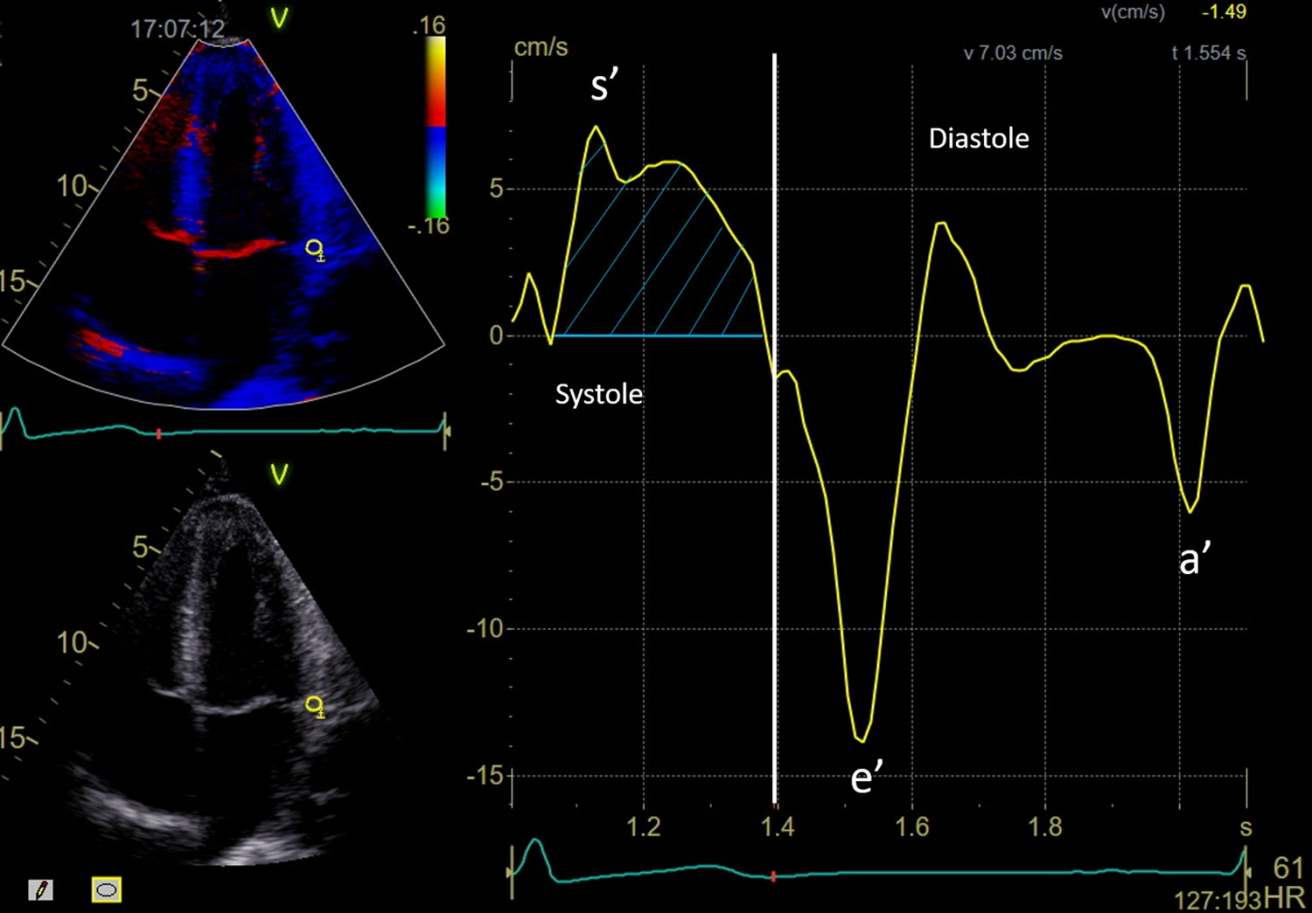
Example of myocardial velocity curve by tissue Doppler imaging. The yellow circle shows the place where the sample is obtained (lateral region of the mitral annulus). Y-axis: myocardial velocity (cm/s). X-axis: time (seconds). The positive peak during systole is marked by s’ on the velocity curve. During diastole, early (e’) and late (a’) diastolic peak is marked. Mitral annular displacement (longitudinal displacement) is calculated from area under the curve during systole (shaded area)

Color TDI loops were obtained in the apical four-chamber, two-chamber, and long-axis views at the highest possible frame rate. Myocardial velocities, including e’, were measured within a 6 mm circular sample volume in the septal, lateral, inferior, anterior, posterior, and anteroseptal mitral annular positions. Longitudinal displacement (LD) was calculated from the area under the velocity curve during ejection using an automated algorithm (Fig. 1). TDI curves were smoothed by averaging myocardial velocities over 30 ms. Both LD and e’ were measured in all of the six basal segments; the averages were calculated and applied in this study.

Previous studies have suggested LD and e’ as being sensitive markers of changes in cardiac function when studying the effect of physical activity. We therefore chose to focus on LD and e’ in the evaluation of myocardial function in our study [19–21].

**Fig. 2:**
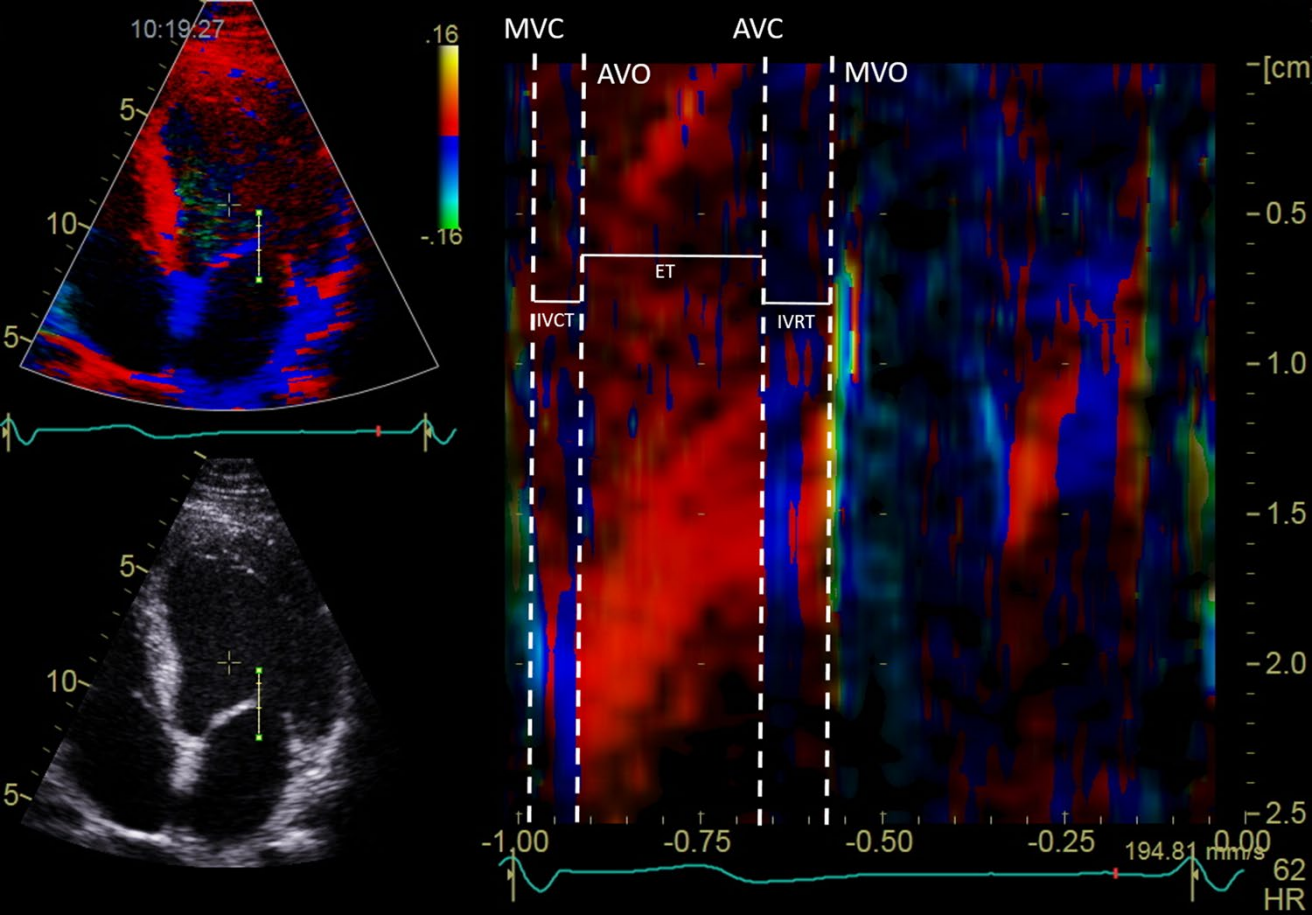
(Left) Four-chamber gray-scale (bottom) and color tissue Doppler imaging (top) views in end-systole showing the position of the M-mode line used for measurement of cardiac intervals. (Right) Color diagram of the tissue Doppler imaging M-mode of the anterior mitral leaflet. *AVC* aortic valve closing, *AVO* aortic valve opening, *MVC* mitral valve closing, *MVO* mitral valve opening, *IVRT* isovolumic relaxation time, *IVCT* isovolumic contraction time, *ET* ejection time

The cardiac time intervals were obtained in the apical four-chamber view at the highest possible frame rate. A 2–4 cm straight M-mode line was placed through the septal half of the mitral leaflet in closed position in end-systole (Fig. 2). Cardiac time intervals were measured based on the color M-mode diagram. The method has been described in detail in a previous publication from the Copenhagen City Heart Study [24].

### Physical activity

All participants filled in the Copenhagen City Heart Study Questionnaires containing questions on previous medical history, lifestyle, and sociodemographics. The physical activity questionnaire included information about activity level both at work and during leisure time. The activity level was scored from 1 to 4 in the questionnaire with increasing activity level (physical activity questionnaire included in the electronic supplementary material). Leisure-time physical activity levels were defined as follows: Group I—almost entirely sedentary, group II—light physical activity for 2 to 4 h per week, group III—light physical activity for more than 4 h per week or more vigorous physical activity for 2 to 4 h per week, and group IV—high vigorous physical activity for more than 4 h per week or regular heavy exercise or competitive sports several times per week. According to the activity level from the questionnaire, participants were divided into four groups: I (inactivity), II (light activity), III (moderate activity), and IV (high-level activity).

### Statistical analyses

For demographics, Pearson’s Chi square test was used for categorical covariates and one-way analysis of variance (ANOVA) for continuous covariates. If the data were not normally distributed (MPI), transformation to natural logarithm was made, and geometric mean was calculated. The Kruskal–Wallis rank test was used instead of ANOVA when data were not normally distributed. Linear regression models in the following age strata were used to examine the associations between activity level and TDI parameters: < 50 years, 50–65 years and > 65 years. Activity level was considered as a categorical variable and TDI measurements were plotted on a continuous scale. In the multivariable analyses, we adjusted for the following pre-specified potential confounders: sex, ischemic heart disease, hypertension, body mass index (BMI), and diabetes. Since resting heart rate (RHR) might affect values of TDI-parameters, we performed a separate analysis where we additionally adjusted for RHR [25]. Interaction analyses were performed to clarify whether interactions between age and physical activity level affected the results. The analyses were performed separately for each TDI parameter. Linearity, variance homogeneity, and the assumption of normality were tested with plots of residuals in each age stratum. P-values < 0.05 on two-sided tests were considered statistically significant. Values in parentheses are 95% confidence intervals unless stated otherwise.

All analyses were performed by STATA Statistics/Data Analysis version 14.1.

## Results

Characteristics of the study population are displayed in Tables 1 and 2. BMI was lower in the highest activity groups. Frequencies of hypertension, diabetes, and ischemic heart disease were lower in higher activity levels. In the highest activity groups, we found higher left atrial volume index (LAVI), and lower resting heart rate (RHR). Left ventricular ejection fraction (LVEF) was similar in all activity groups.

**Table 1:** Baseline characteristics of study population in relation to level of physical activity

	The total population (N = 2053)	Inactivity (N = 180)	Light activity (N = 1016)	Moderate activity (N = 753)	High level activity (N = 104)	p-value
Age, years	58.3 ± 16.0	60.6 ± 16.8	60.8 ± 15.5	55.8 ± 15.3	48.0 ± 18.6	< 0.01
Male sex, %	42.7	48.3	37.7	46.1	56.7	< 0.01
Body Mass Index, kg/m^2^	25.5 ± 3.9	26.3 ± 4.4	25.7 ± 4.1	25.0 ± 3.6	24.9 ± 3.2	< 0.01
Resting HR/bpm	67.1 ± 11.3	70.0 ± 10.8	67.6 ± 11.3	66.3 ± 10.9	62.1 ± 12.4	< 0.01
Hypertension, %	43.3	55.3	47.6	36.8	26.9	< 0.01
Diabetes mellitus, %	10.1	11.2	11.2	9.0	3.9	< 0.01
IHD, %	13.9	19.4	16.4	10.2	5.8	< 0.01
Smoking, %						< 0.01
Never smoked	32.4	23.9	31.3	35.2	37.5	
Current smoker	33.4	50.6	35.9	27.0	27.9	
Former smoker	33.5	25.6	32.2	37.5	32.7	
LVEF, %	59.7 ± 2.1	59.8 ± 1.1	59.6 ± 2.6	59.8 ± 1.4	60.0 ± 0.4	NS
LAVI, ml/m^2^	19.3 ± 6.5	17.9 ± 5.5	19.4 ± 7.3	19.3 ± 5.6	21.3 ± 5.8	< 0.01
e’ (cm/s)	7.2 ± 2.7	6.7 ± 2.7	6.8 ± 2.6	7.5 ± 2.6	8.8 ± 3.3	< 0.01
LD (mm)	10.6 ± 2.3	10.4 ± 2.1	10.3 ± 2.2	10.8 ± 2.2	11.6 ± 2.5	< 0.01
MPI (median, in parenthesis: 25% and 75% percentiles)	0.47 (0.40–0.56)	0.48 (0.43–0.55)	0.48 (0.41–0.56)	0.47 (0.39–0.55)	0.44 (0.39–0.51)	< 0.01

*HR* heart rate/ beats per minute, *IHD* ischemic heart disease, *LVEF* left ventricular ejection fraction, *LAVI* left atrial volume index, *LD* longitudinal displacement, *MPI* Myocardial Performance Index, MPI = (IVCT + IVRT)/ET, *IVCT* isovolumic contraction time, *IVRT* isovolumic relaxation time. Mean and standard deviations, unless specified otherwise. *NS* non-significant

**Table 2:** Number of persons in each age stratum and activity group

	Age < 50 years	Age 50–65 years	Age > 65 years	In total
I: Inactivity	53	47	80	180
II: Light activity	263	318	435	1016
III: Moderate activity	261	269	223	753
IV: High activity	57	25	22	104
In total	634	659	760	2053

To adjust for confounding concerning age, the population was divided into three age strata when we analyzed the tissue Doppler parameters: <50 years, 50–65 years, and > 65 years.

### Cardiac function and physical activity

**Fig. 3:**
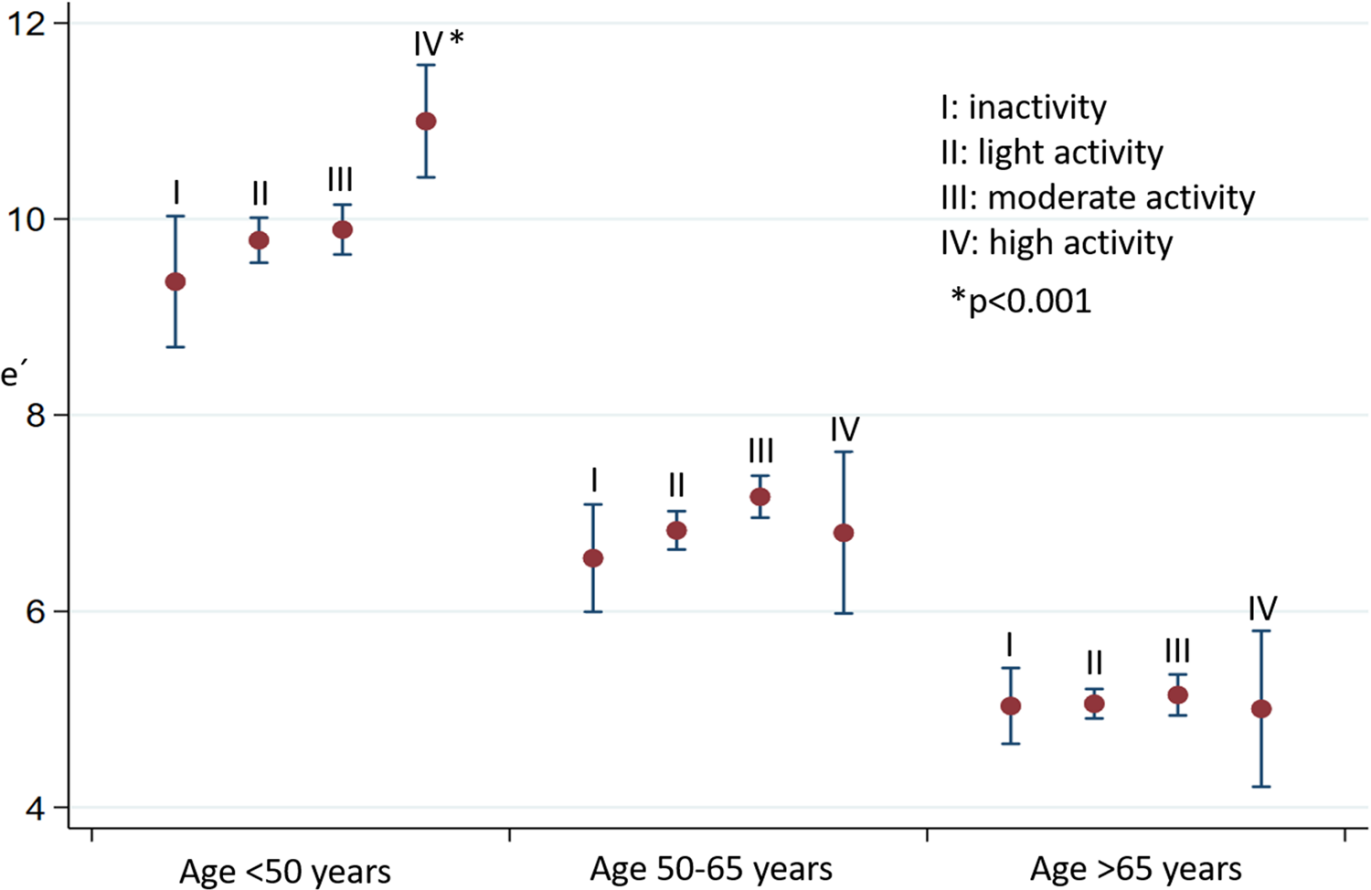
Early diastolic velocity e’ in relation to age group and activity level. Mean and 95% confidence intervals

**Fig. 4:**
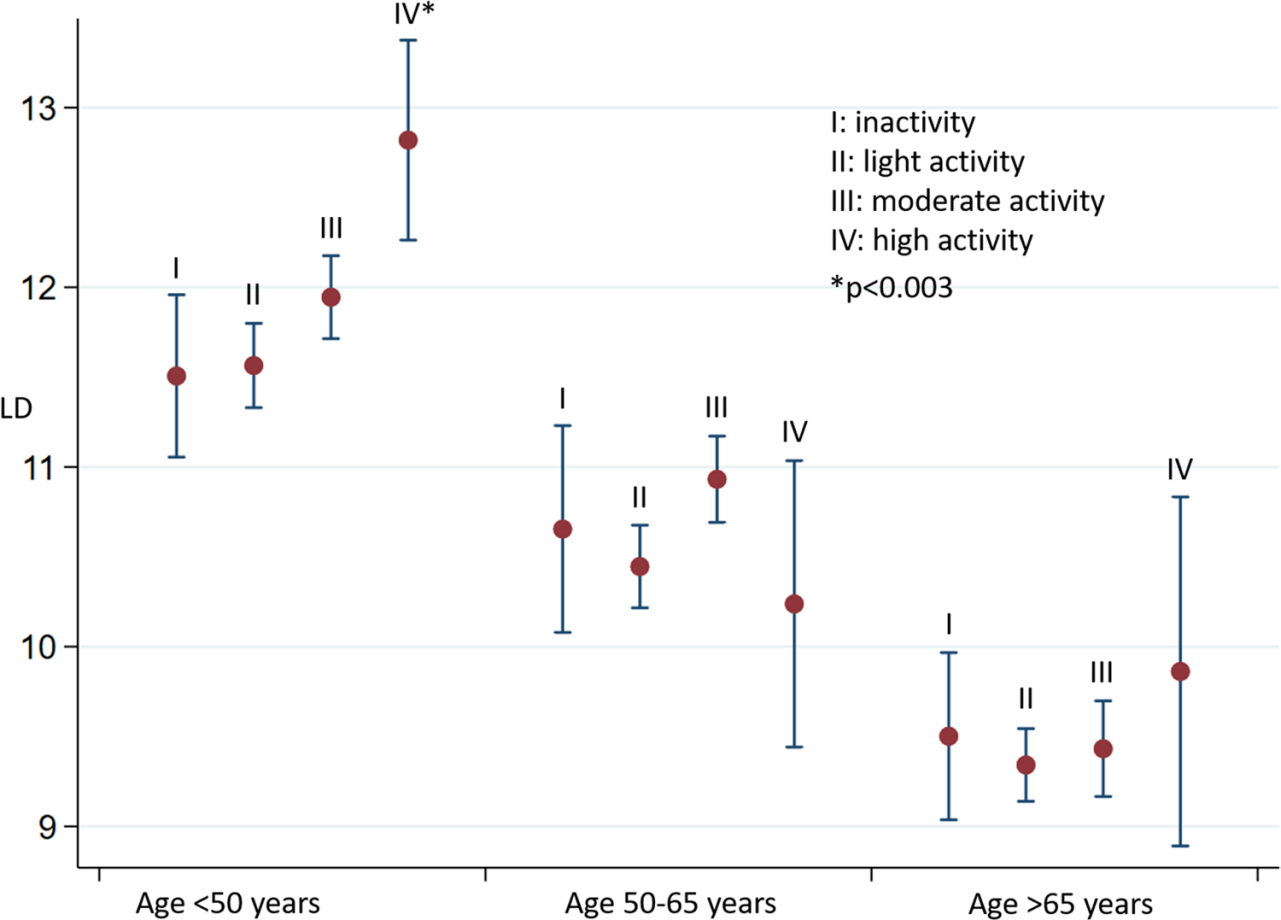
Longitudinal displacement (LD) in relation to age group and activity level. Mean and 95% confidence intervals

**Fig. 5:**
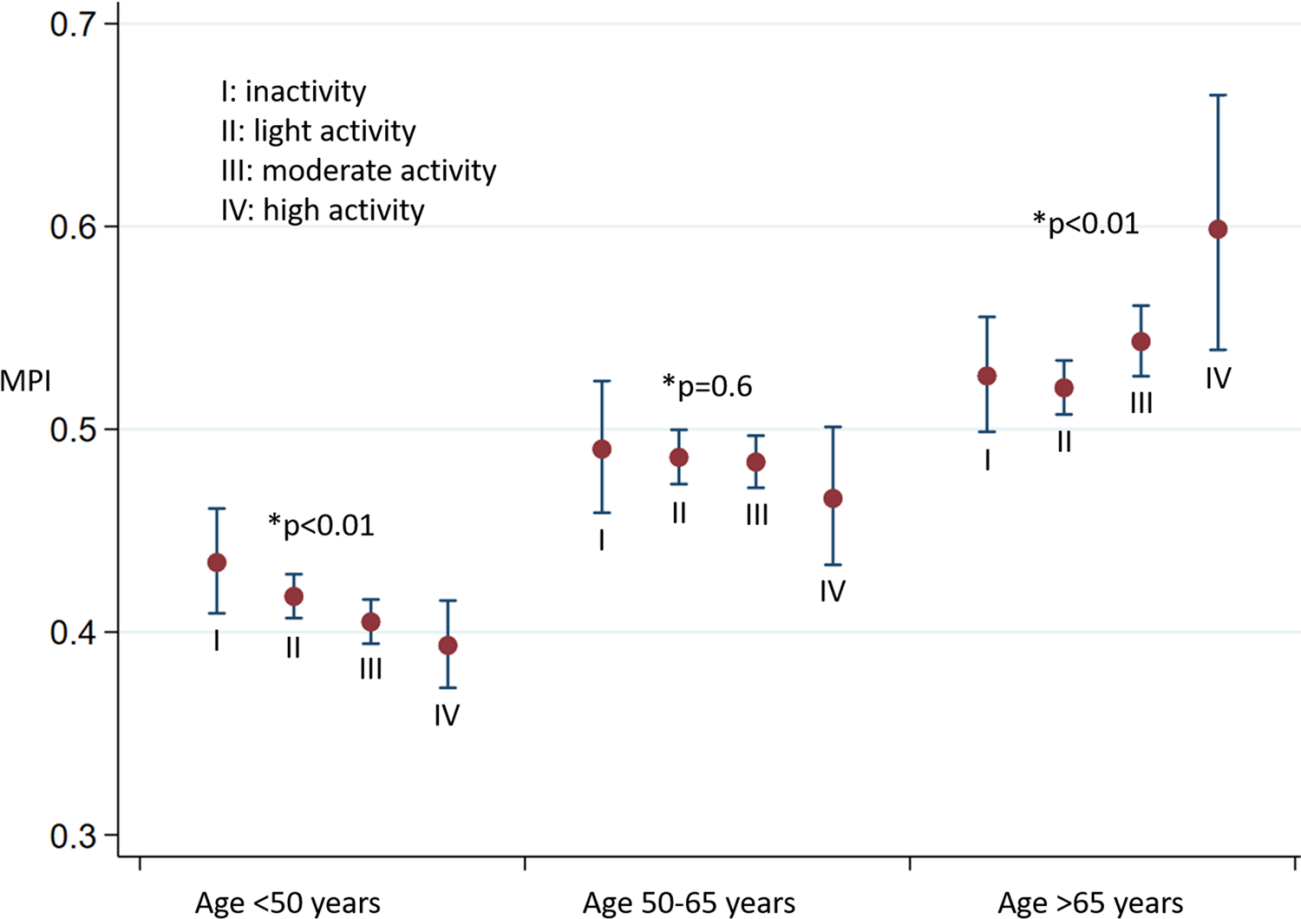
MPI (geometric mean and 95% confidence intervals) in relation to age group and activity level. MPI= (IVRT + IVCT)/ET. *MPI* myocardial performance index, *IVRT* isovolumic relaxation time, *IVCT* isovolumic contraction time, *ET* ejection time. *The p-values in trend analysis for MPI within each age group

Linear regression analysis was performed in each age group to clarify whether the level of physical activity was associated with myocardial performance. Figures 3, 4, and 5 illustrate the associations between activity level and cardiac function in each age group.

All of the echocardiographic parameters e’, LD, and MPI were strongly associated with age, regardless of activity levels. In the following, we describe the associations within each age group.

#### Age < 50 years

In this age group, we found improved systolic and diastolic function with increased activity level. Both LD and e’ were significantly higher in high level activity (group IV) compared to the three other activity levels. This pattern remained significant after adjusting for sex, diabetes, hypertension, BMI, and ischemic heart disease (e’ p < 0.001, LD: p < 0.003, MPI: p = 0.02). Even after Bonferroni correction, activity group IV had a significantly higher values of LD and e’. Trend tests were made with activity level as a continuous variable. Here, we found that activity levels were significantly associated with improved cardiac function (higher e’ (p = 0.001), LD (p = 0.004) and lower MPI (p < 0.002)). The decrease in MPI was primarily due to a decrease in IVRT/ET (p < 0.001) in higher activity levels.

#### Age 50–65 years

In this age group, we did not find any significant association between level of physical activity and systolic or diastolic myocardial performance (e’ p = 0.17; LD: p = 0.16; IVRT/ET: p = 0.92; IVCT/ET: p = 0.83; MPI: p = 0.58).

#### Age > 65 years

In age > 65 years, both systolic and diastolic myocardial function seemed to be similar in all four activity levels when activity levels were analyzed as a categorical variable. The trend test (activity levels as a continuous variable) revealed a significant tendency towards reduced myocardial function with increased activity level independent of sex, ischemic heart disease, hypertension, BMI, and diabetes (an increase of MPI by 0.02 (0.006–0.038) for each higher activity level, p < 0.01). No significant changes in e’ and LD were found (e’: p = 0.402; LD: p = 0.452).

#### Adjustment for RHR

We performed separate analyses where we additionally adjusted for RHR. Adjusting for RHR did not change any of the results.

### Interaction analyses

Interaction analyses with adjustment for potential confounders revealed interaction between age group and activity level when studying e’ (p < 0.008). This means that the association between cardiac function and activity level is changed by aging; physical activity in young age was associated with higher levels of e’ compared with the older age groups. For LD, we did not find any significant interaction (p = 0.182).

Interaction analyses revealed a significant interaction between activity level and age when studying MPI (p < 0.001).

## Resting heart rate and level of physical activity

**Fig. 6:**
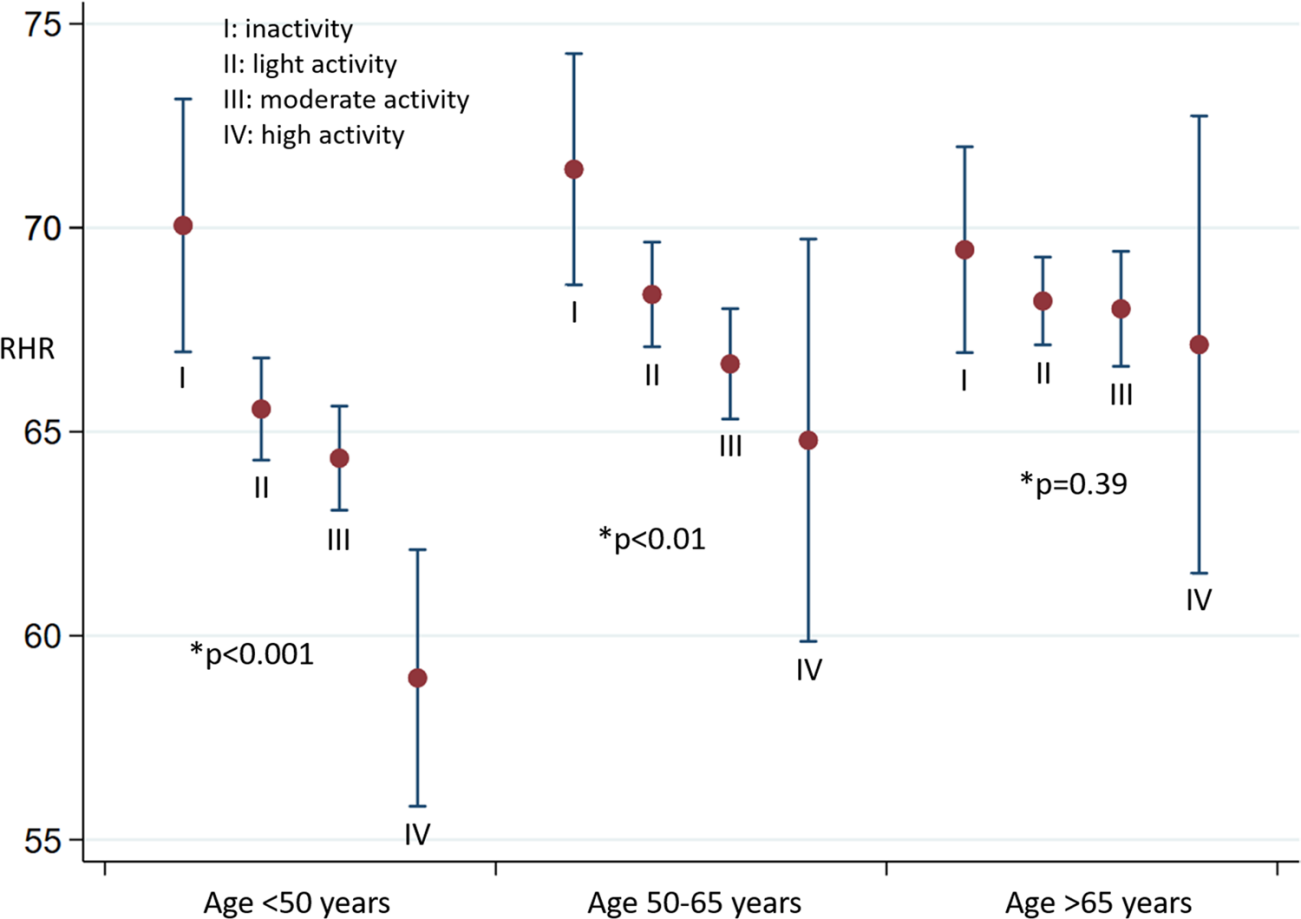
Resting heart rate (RHR, beats per minute) in relation to age group and activity level. *The p-values in trend analysis for RHR within each age group

We studied the RHR in relation to age group and level of physical activity (Fig. 6). There was a tendency towards a lowering of the RHR in higher activity levels when studying age groups < 50 years, and 50–65 years. For individuals aged < 50 years, we found a drop in heart rate of 2.4 beats/min (95% CI 0.7–3.6) for each increased level of activity (p < 0.001). A decrease of 2.0 beats/min (95% CI 0.7–3.4) was found for trend analysis in persons aged 50–65 years (p < 0.01). Both findings were statistically significant. For persons aged > 65 years, we did not find any association between the level of physical activity and the heart rate (p = 0.39).

## Discussion

This large community-based study investigated the association between cardiac function and level of physical activity. To the best of our knowledge, this is the first study to examine this association in relation to age. Our results indicate that a better myocardial performance in relation to higher levels of physical activity is achieved only when age is below 50 years. In age > 65 years, there seems to be a tendency for impairment of myocardial performance at higher activity levels.

The parameter e’ is a sensitive and frequently used echocardiographic measure in studies on the diastolic function in relation to physical activity including sports [20, 26–28]. Moreover, e’ has been shown to be significantly higher in endurance and strength athletes. LD is associated with stroke volume and ejection fraction [29–31]. The studies including LD as a measure of systolic function found significant changes in relation to physical activity [19, 20].

Studies of the athlete’s heart have revealed structural and functional changes towards higher systolic and diastolic performance assessed by TDI when compared with age-matched non-athletes [26–28]. Usually, athletes belong to a young age group. Our results support these findings; thus we also find a high level of physical activity to be associated with improvement in myocardial performance in the younger age group.

Andersen et al. described improved systolic and diastolic myocardial function in sedentary women less than 50 years of age (36.5 ± 8.2 years) undergoing 16 weeks of regular physical training [20]. These findings suggest that cardiac adaptations are easily achieved in the younger age group. However, changes in cardiac function in older age groups in relation to physical activity require further investigation.

A few studies have investigated the association between cardiac time intervals and the level of physical activity. In 1999, Libonati et al. described how systolic time intervals were prolonged and isovolumic relaxation time shortened in long-distance runners in comparison to short-distance runners [32]. They also found lower levels of MPI in long-distance runners. Seismocardiography was used to determine the cardiac time intervals, which is different from the method we used in our study. Despite differences in the study population, our study confirms the findings by Libonati et al.

From a physiological point of view, it is reasonable to assume that a younger heart might be more sensitive to changes in myocardial performance in relation to exercise. One of the reasons could be that this age group has a low frequency of medical conditions that affect cardiac function such as hypertension, diabetes, and IHD. In addition, aging is associated with molecular and cellular alterations including increased interstitial myocardial fibrosis [33–35]. These changes cause altered myocyte contractile function, stiffness of the myocardium, and thus impairment of systolic as well as diastolic function in the aging population.

The interaction analyses in our study show that age might be an effect modifier when studying the association between physical activity and myocardial performance. These findings emphasize the importance of age when studying cardiac adaptations in relation to physical activity. Effect modification in this context means that younger age groups have a greater impact on improving cardiac function when exercising. In age groups < 50 years and 50–65 years, the resting heart rate decreased in higher levels of activity, whereas the heart rate was unchanged in those aged > 65 years. A decrease in resting heart rate during physical activity is a basic physiological change. In our study population, this correlation was seen only in those aged < 65 years. This is in line with the findings by TDI, revealing that improved myocardial performance was associated with increasing levels of activity in age groups < 65 years, but not in those aged > 65 years.

Our results raise the question of whether myocardial performance is reversed by aging regardless of exercise. There may be a cut-off point in age, a “point of no return,” where the beneficial effects of physical activity on myocardial performance might no longer exist.

One of the limitations of our study is that we do not have data for how many years the participants have had the reported physical activity level, and whether their activity level changed during their lifetime. We assume that the reported activity level has been the most dominant level during their adult lifetime. Thus, we assume that the participants in the two highest age groups have had a high level of physical activity for many years, if reported as such.

In defining the groups according to their level of activity, we primarily focused on the self-reported activity level in leisure time. In 792 persons, we did not have data on their activity level during working hours, so the analyses could not be performed with classification of activity levels at work only.

However, in this study, physical activity was not an intervention, and the results reflect associations, and no causal inference can be assessed. Previous studies on physical exercise as an intervention reported improvement in cardiac function measured by TDI. More studies of this kind are needed, especially studies including persons > 50 years of age.

Previous studies comparing aerobic interval training with continuous endurance training found better myocardial performance after interval training [20]. How aging affects this association thus requires further exploration.

The current recommendations concerning regular physical activity do not distinguish between differences in age. Even though several studies have shown a reduction in cardiovascular disease and mortality in those who are physically active, the effects on myocardial performance are sparsely studied. It is scientifically interesting whether a reduction in mortality and cardiovascular disease in relation to physical activity in the general population is associated with improved myocardial performance. Schnohr et al. found that long-term moderate or high levels of physical activity were associated with significantly lower all-cause mortality and death from coronary heart disease in an observational study design [4]. Physical activity induces physiological benefits such as increased insulin sensitivity, lower blood pressure, and improved lipid profile, which could partly explain these findings. However, persons capable of participating in moderate to high levels of physical activity have good physical health, so the increased survival time for these individuals might therefore be explained by factors other than only physical activity.

Our study results reveal that aging could reverse the benefits of physical activity on cardiac function. These findings need to be confirmed in future studies.

## Conclusion

In the general population, the association between increased levels of physical activity and improved cardiac function, assessed by TDI, was found only in persons < 50 years of age. In persons aged > 65 years, higher levels of physical activity were associated with reduced myocardial performance. Our results suggest that the beneficial associations between myocardial performance and physical activity are altered and reversed by aging. Myocardial performance measured by TDI decreases with age, independent of the level of physical activity. Additional studies are needed to explore more associations between physical activity and myocardial performance in relation to age and exercise modalities and training protocols.

## Electronic supplementary material

Below is the link to the electronic supplementary material.

Supplementary material 1 (DOCX 17 KB)

## Abbreviations

BMI: Body mass index
ET: Ejection time
IHD: Ischemic heart disease
IVCT: Isovolumic contraction time
IVRT: Isovolumic relaxation time
LAVI: Left atrial volume index
LD: Longitudinal displacement
LVEF: Left ventricular ejection fraction
MPI: Myocardial performance index
RHR: Resting heart rate
TDI: Tissue Doppler imaging
