# Supplementary material for: The association between physical activity and cardiac performance is dependent on age: the Copenhagen City Heart Study
Source: Int J Cardiovasc Imaging. 2019 Mar 1;35(7):1249–58. doi: 10.1007/s10554-019-01566-0 (PMC6598956; doi:10.1007/s10554-019-01566-0)
Supplement: Supplementary file 1 — Supplementary material 1 (DOCX 17 KB) [file 10554_2019_1566_MOESM1_ESM.docx]

**Electronic supplementary material**

The association between physical activity and cardiac performance is dependent on age: The Copenhagen City Heart Study

**International Journal of Cardiovascular Imaging**

*Gowsini Joseph MD *, Rasmus Møgelvang MD, PhD, Tor Biering-Sørensen MD, PhD, Gitte Nielsen MD, PhD, Peter Schnohr MD, DMSc, Peter Søgaard MD, DMSc*

***Corresponding author: Gowsini Joseph, Department of Clinical Medicine, Aalborg University, Aalborg, Denmark, email:** [**gowsini@gmail.com**](mailto:gowsini@gmail.com)

**Supplementary material**

1. **The physical activity questionnaire**

Information on activity level at work as well as in leisure time was included. The activity level was scored from 1 to 4 in the questionnaire with increasing activity level.

***Question 1***

Please indicate your PHYSICAL ACTIVITY LEVEL DURING WORK over the last year (students, home-makers and the unemployed should fill out the answers while retirees without work should proceed to question 2) (choose one option only)

| I | Primarily seated while working  e.g. deskwork, home-maker with no children and with domestic help |  |
| --- | --- | --- |
| II | Sitting or standing, sometimes walking  e.g. clerk, teacher, home-maker washing and cleaning without small children |  |
| III | Walking, lifting once in a while  e.g. postal worker, caretaker, home-maker washing and cleaning with one or more small children |  |
| IV | Heavy physical work  e.g. construction worker, furniture remover |  |

If options III or IV were chosen. Do you often lift heavy weights? YES ____ NO ____

***Question 2***

Please indicate your PHYSICAL ACTIVITY LEVEL IN YOUR SPARE TIME (including transportation to and from work) over the last year (choose one option only)

| I | Almost completely physically passive or physically active for less than 2 hours per week  e.g. reading, watching TV, going to the cinema |  |
| --- | --- | --- |
| II | Light physically activity 2-4 hours per week  e.g. walking, cycling, light garden work or low-intensity workouts |  |
| III | Light physical activity more than 4 hours per week or more strenuous activity 2-4 hours per week  e.g. fast walking and/or fast cycling, heavy garden work, high-intensity workouts involving getting sweaty and short of breath |  |
| IV | More strenuous physical activity for more than 4 hours per week or regular high-intensity workouts or sporting competitions several times per week |  |
